# Simplified Decision-Tree Algorithm to Predict Falls for Community-Dwelling Older Adults

**DOI:** 10.3390/jcm10215184

**Published:** 2021-11-05

**Authors:** Keitaro Makino, Sangyoon Lee, Seongryu Bae, Ippei Chiba, Kenji Harada, Osamu Katayama, Kouki Tomida, Masanori Morikawa, Hiroyuki Shimada

**Affiliations:** 1 Department of Preventive Gerontology, Center for Gerontology and Social Science, National Center for Geriatrics and Gerontology, 7-430 Morioka-cho, Obu City 474-8511, Japan; sylee@ncgg.go.jp (S.L.); bae-sr@ncgg.go.jp (S.B.); ichiba@ncgg.go.jp (I.C.); harada-k@ncgg.go.jp (K.H.); katayama.o@ncgg.go.jp (O.K.); tomida-k@ncgg.go.jp (K.T.); morikawa@ncgg.go.jp (M.M.); 2 Research Fellowship for Young Scientists, Japan Society for the Promotion of Science, Chiyoda-ku, Tokyo 102-0083, Japan; 3 Center for Gerontology and Social Science, National Center for Geriatrics and Gerontology, 7-430 Morioka-cho, Obu City 474-8511, Japan; shimada@ncgg.go.jp

**Keywords:** fall prevention, decision-tree, machine learning, risk prediction

## Abstract

The present study developed a simplified decision-tree algorithm for fall prediction with easily measurable predictors using data from a longitudinal cohort study: 2520 community-dwelling older adults aged 65 years or older participated. Fall history, age, sex, fear of falling, prescribed medication, knee osteoarthritis, lower limb pain, gait speed, and timed up and go test were assessed in the baseline survey as fall predictors. Moreover, recent falls were assessed in the follow-up survey. We created a fall-prediction algorithm using decision-tree analysis (C5.0) that included 14 nodes with six predictors, and the model could stratify the probabilities of fall incidence ranging from 30.4% to 71.9%. Additionally, the decision-tree model outperformed a logistic regression model with respect to the area under the curve (0.70 vs. 0.64), accuracy (0.65 vs. 0.62), sensitivity (0.62 vs. 0.50), positive predictive value (0.66 vs. 0.65), and negative predictive value (0.64 vs. 0.59). Our decision-tree model consists of common and easily measurable fall predictors, and its white-box algorithm can explain the reasons for risk stratification; therefore, it can be implemented in clinical practices. Our findings provide useful information for the early screening of fall risk and the promotion of timely strategies for fall prevention in community and clinical settings.

## 1. Introduction

Falls are a major public health problem, and approximately 28–35% of individuals aged ≥65 years fall each year [1]. Fall-related injuries are associated with disability [2] and mortality [3]; the fall-associated financial costs, including those of ambulance services and health and social care, are substantial and continuously increasing worldwide [1]. Therefore, early screening of fall risk is necessary to promote effective fall prevention strategies.

Previous research has revealed several fall risk factors, such as previous fall history [4], gait and balance impairments [4,5], arthritis [4], pain [4], polypharmacy [5,6], and fear of falling (FOF) [7]; thus, multifactorial risk assessment is often recommended [8]. The American Geriatrics Society and British Geriatrics Society (AGS/BGS) Panel has published clinical practice guidelines for the prevention of falls in older persons and provided a conceptual algorithm with multiple risk factors for assessment and intervention to reduce the frequency of falls in older adults [9]. However, statistical examination of decision-making algorithms for fall prediction, with respect to hierarchy, or optimal combination of risk assessment have not been fully considered.

Recently, machine learning methods that can iteratively learn nonlinear interactions from large samples using computer algorithms have been applied in various fields, including disease risk assessment and prediction [10]. In particular, decision-tree analysis can provide an intuitive diagram that represents risk prediction without the need for complicated calculations [11]. Thus, decision-tree analysis has been used in many fields for decision-making purposes to develop models that can classify subjects into various risk categories [12]. 

We identified several previous studies that have examined the utility of the decision-tree model in predicting falls in community-dwelling older adults [13,14,15]. Stel et al. created a decision-tree model to predict recurrent falls based on known risk factors (e.g., fall history, physical performance, pain, physical activity, and limitation in activities of daily living) and showed that the risk of recurrent falls could be stratified by 9–70% [13]. However, they did not report their performance measures, such as accuracy or area under the curve (AUC), because they did not validate their model with another dataset. Gomez et al. and Lam et al. also proposed fall-prediction models that included multiple risk factors based on a decision-tree analysis, with performance measures for community-dwelling older adults [14,15]. However, their prediction variables included the scores of test batteries (i.e., a short physical performance battery or frailty criteria), which have already been combined with multiple assessment items. Some recent studies have attempted to improve predictive accuracy by using ensemble methods, which create many (hundreds) decision trees while predictions from each tree are aggregated. Speiser et al. developed a prediction model for serious fall injury using random forest method; the authors achieved a prediction accuracy higher than that of a single decision tree model [16]. Ye et al. developed a fall prediction model using extreme gradient boosting with electronic health records to achieve high performance (C-statistic = 0.81) [17]. Although these ensemble methods provide a relatively high prediction accuracy, they have the disadvantage of making it difficult to visually interpret or explain the results. Therefore, there is still opportunity to examine the minimum and optimal combinations of fall predictors consisting of common and easily measurable items, and it is worthwhile to illustrate the results as a single decision tree.

This study aimed to develop a simplified decision-tree algorithm for fall prediction using easily measurable predictors with longitudinal cohort data. We hypothesized that the decision-tree model would predict falls more accurately than a logistic regression model.

## 2. Materials and Methods

### 2.1. Design

In this longitudinal observational study, fall predictors for community-dwelling older adults were assessed in a baseline survey (August 2011 to February 2012) and recent falls were assessed in a follow-up survey (August 2015 to February 2016). The participants were enrolled from a sub-cohort of the National Center for Geriatrics and Gerontology–Study of Geriatric Syndromes, a population-based national cohort study. 

All assessments were conducted by trained nurses and study assistants at community centers. Before the study began, we trained all the staff regarding the appropriate protocols for conducting these assessments.

### 2.2. Participants

**Figure 1:**
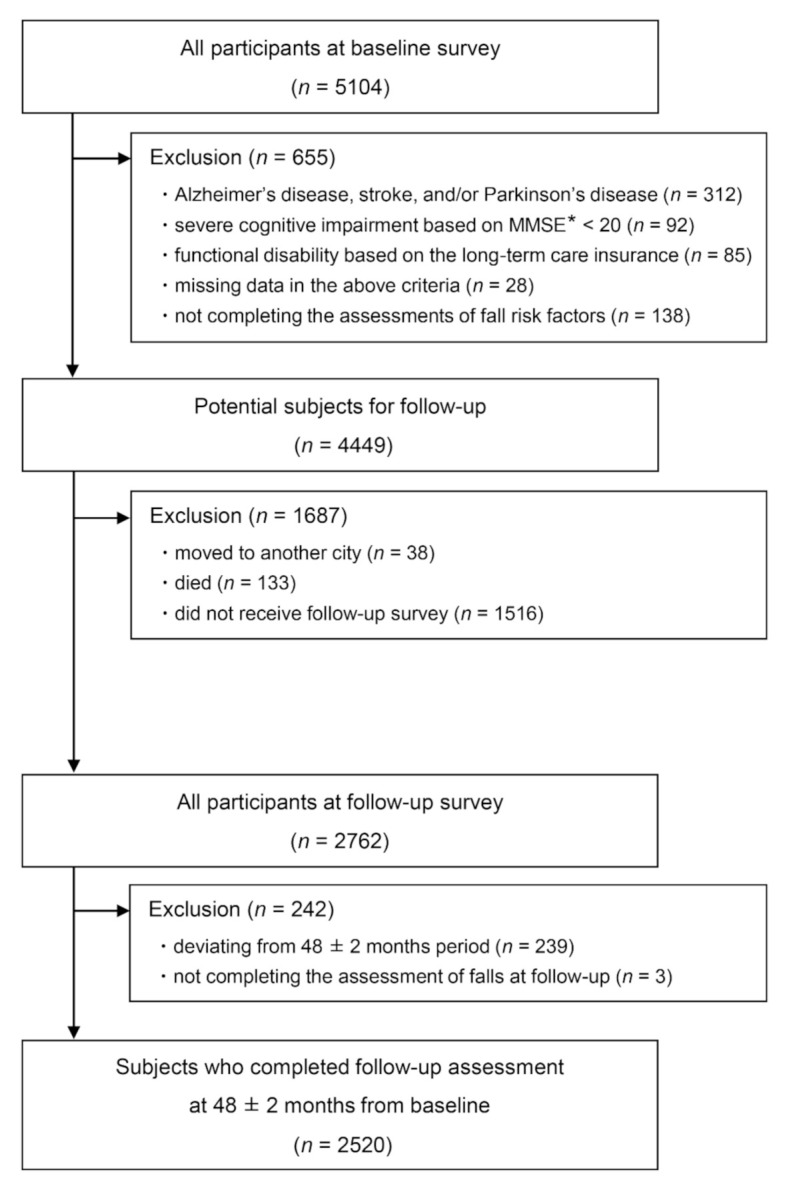
Flowchart of participant recruitment and screening. * MMSE, Mini-Mental State Examination.

Individuals aged 65 years or older who lived in Obu, Japan, were invited to participate in this study. We applied the following exclusion criteria in the baseline survey: (1) history of Alzheimer’s disease, stroke, or Parkinson’s disease; (2) severe cognitive impairment based on the mini-mental state examination [18] score that was less than 20; (3) certification by the national long-term care insurance system as having a functional disability; (4) missing data for these criteria; and (5) lack of assessment of fall risk factors. After the exclusions, the candidate subjects were invited to complete a follow-up survey 48 ± 2 months after the baseline survey. During the follow-up period, we excluded those participants who (1) had moved to another city, (2) had died, or (3) did not receive the follow-up survey. After the follow-up survey, we also excluded the following participants: (1) those deviating from the 48 ± 2 months follow-up period and (2) those who did not complete the fall assessment in the follow-up survey. After exclusions, data from 2520 participants were analyzed (Figure 1).

Written informed consent was obtained from all participants prior to their inclusion in the study. This study was conducted in accordance with the Declaration of Helsinki, and the ethics committee of the National Center for Geriatrics and Gerontology approved the study protocol (approval number: 1440-2).

### 2.3. Assessment of Falls

Falls were assessed by face-to-face interviews in both baseline (fall history) and follow-up (primary outcome) surveys. A fall was defined as “an unexpected event in which a person comes to rest on the ground, floor, or a lower level” [19]. A recent fall was measured by participants’ responses to the following question: “Do you have any history of a fall within the past year?” [20] In this study, fall history was defined as at least one fall within the past year in the baseline survey, and at least one fall within the past year in the follow-up survey was used as the outcome of the decision-tree algorithm [21,22]. 

### 2.4. Assessment of Fall Predictors

As fall-prediction variables, we assessed common fall risk factors as well as fall history, age, sex, FOF, prescribed medication, knee osteoarthritis, lower limb pain, gait speed, and timed up and go test (TUG). FOF was assessed by a closed-ended question: “Are you afraid of falling?” [23]. Participants who selected “very much” or “somewhat” were classified as having FOF, whereas participants who chose “a little” or “not at all” were classified as those without FOF [24]. Prescribed medication was assessed as the total number of all drugs continuously prescribed by a doctor to the individual, and we defined ≥5 drugs as polypharmacy [6]. Medical history of knee osteoarthritis and presence of daily pain in the lower limbs were assessed through face-to-face interviews. Regarding physical performance tests, the AGS/BGS guideline states that fall risk assessments should include gait and balance evaluation [9]; therefore we measured TUG that is recommended in the guideline in addition to gait speed [9]. Gait speed was measured in five trials using a stopwatch. Participants were asked to walk on a flat and straight surface at a comfortable gait speed. Two markers were used to indicate the start and end of a 2.4-m walk path, with a 2-m section to be traversed before passing the start marker so that participants were walking at a comfortable pace by the time they reached the timed section. Participants were asked to walk a further distance of 2-m past the end of the path to ensure a consistent walking pace while on the timed path [25]. In our gait speed measurement protocol, a relatively short walking path was set; therefore, a preliminary experiment was conducted to confirm the correlation between 10-m and 2.4-m gait speeds (r = 0.989, *p* < 0.01) [26]. The mean gait speed from the five trials was used as a fall-prediction variable in this study. The TUG time was measured as the time taken to rise from a standard armchair, walk a distance of 3 m at a normal and safe pace, turn around, walk back to the chair, and sit down again [27]. A previous study demonstrated that TUG had a high reliability (intraclass correlation coefficient (3,3) = 0.98) and could identify fallers (accuracy = 0.87) [28]. Two trials of TUG were conducted, and the mean time to complete the test was used as a prediction variable in this study.

### 2.5. Statistical Analysis

First, we classified the participants as fallers or non-fallers according to fall status in the follow-up survey and compared their baseline characteristics using Student’s *t*-test for continuous variables and χ^2^ test for categorical variables. We also calculated the odds ratios and 95% confidence intervals of all potential predictors assessed in this study. Second, we created fall-prediction models. In this procedure, a random resampling technique was applied in the minor class (fallers) and the imbalanced data were corrected into balanced data (fallers:non-fallers = 1:1) because some supervised algorithms with imbalanced datasets deliver inferior performance [29]. Next, we performed a decision-tree analysis using the C5.0 algorithm to identify the optimal and minimum combination of risk factors necessary to predict the fall status in the follow-up survey. The C5.0 algorithm is a classification approach that generates a tree in a top-down scheme based on the provided information using a recursive process [30]. In the process of building the decision tree, the optimal cut-off point (threshold at which the fall risk can be most clearly classified) is automatically calculated as a branch for continuous variables. To improve the model’s performance, we generated 100 boosted decision trees. We conducted global pruning with 75% pruning severity to avoid overfitting. The minimum node size was set at 100. Furthermore, 10-fold cross-validation [31] was performed to test the stability of the decision tree. We also created a logistic regression model as a benchmark to evaluate the decision-tree model. This logistic regression analysis was performed via a backward stepwise approach using the same prediction variables as those in the decision-tree analysis. Finally, we identified the model performance of the decision-tree model and logistic regression model using AUC, based on the receiver operating characteristic analysis, accuracy, sensitivity, specificity, positive predictive value (PPV), and negative predictive value (NPV). All analyses were performed using IBM SPSS Statistics 25 and IBM SPSS Modeler 18 (IBM Japan, Tokyo, Japan). The level of statistical significance was set to *p* < 0.05.

## 3. Results

### 3.1. Flow of Participants and Their Characteristics

A total of 2520 community-dwelling older adults aged 65 years or older met our criteria, and their longitudinal data were analyzed. Among the 2520 participants enrolled in this study, 415 (16.5%) reported recent falls in the 48-month follow-up survey. The differences in baseline characteristics between fallers and non-fallers are shown in Table 1. Compared to non-fallers, fallers were significantly older (P = 0.001), had a higher prevalence of fall history (*p* < 0.001) and FOF (*p* < 0.001), took more prescribed medication (*p* = 0.002), had a higher prevalence of knee osteoarthritis (*p* = 0.005) and lower limb pain (*p* = 0.015), and showed slower gait speed (*p* < 0.001) and TUG time (*p* = 0.007).

### 3.2. Prospective Association between Potential Predictors and Future Falls

Odds ratios (ORs) and 95% confidence intervals (95% CIs) of all potential fall predictors in the follow-up survey are shown in Table 2. All the potential predictors, except for sex, were significantly associated with falls in the crude model, and the ORs (95% Cis) of each predictor were as follows: age group (≥75 years): 1.59 [1.26–2.01], sex (female): 1.21 (0.98–1.50), fall history (yes): 3.16 (2.44–4.09), FOF (yes): 1.59 [1.29–1.97], polypharmacy (yes): 1.88 (1.37–2.57), knee osteoarthritis (yes): 1.49 (1.13–1.98), lower limb pain (yes): 1.36 (1.06–1.73), gait speed (m/s): 0.31 (0.18–0.53), and TUG (s): 1.10 (1.03–1.18).

### 3.3. Fall-Prediction Models Using Decision-Tree and Logistic Regression

**Figure 2:**
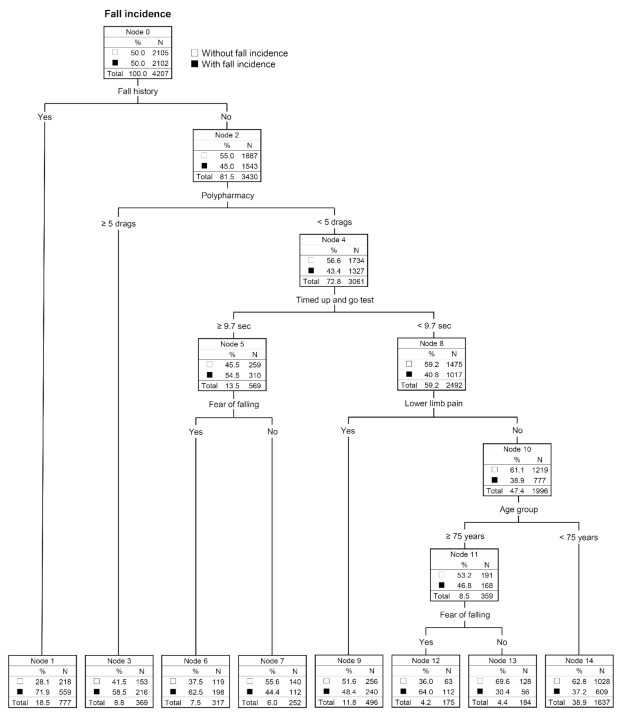
Decision-tree model for fall prediction.

**Figure 3:**
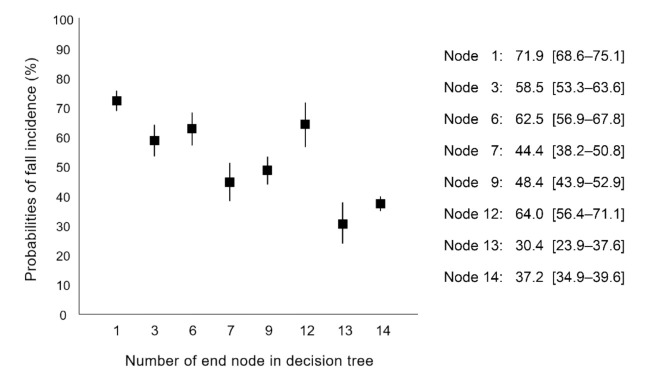
Stratified fall risk in the 8 terminal nodes of decision-tree model. The data are expressed as fall probabilities with 95% confidence intervals.

The final decision-tree model is shown in Figure 2. This model includes 14 nodes with six predictors as follows: fall history, polypharmacy, TUG, FOF, lower limb pain, and age group. The decision tree subdivided the samples into eight risk groups with fall incidence probabilities ranging from 30.4% to 71.9% (Figure 3). We also performed multivariable logistic regression analysis using a backward stepwise approach, and the final logistic regression model is shown in Table 3. Six predictors were selected in the final model, with the following ORs (95% Cis): age group (≥75 years): 1.28 (1.10–1.50), fall history (yes): 2.92 (2.46–3.47), FOF (yes): 1.37 (1.21–1.56), polypharmacy (yes): 1.64 (1.34–2.01), knee osteoarthritis (yes): 1.26 (1.05–1.50), and gait speed (m/s): 0.55 (0.39–0.76).

### 3.4. Comparison of Model Performance

The performances of the logistic regression and decision-tree models are shown in Table 4. AUC, accuracy, sensitivity, specificity, PPV, and NPV were 0.64, 0.62, 0.50, 0.73, 0.65, and 0.59, respectively, in the logistic regression model and 0.70, 0.65, 0.62, 0.69, 0.66, and 0.64, respectively, in the decision-tree model.

## 4. Discussion

We aimed to develop a simplified decision-tree algorithm for fall prediction using easily measurable predictors and examine prediction validity using longitudinal cohort data. We created a decision-tree model that uses six predictors that are common and easily measurable items, and the model could stratify the probabilities of fall incidence ranging from 30.4% to 71.9%. Additionally, the decision-tree model outperformed the logistic regression model with respect to AUC, accuracy, sensitivity, PPV, and NPV.

Regarding the components of our decision-tree model, previous studies have demonstrated the association of fall history [4], polypharmacy [5,6], TUG [28], FOF [7], pain [4], and age group [4] with falls in older people; thus, the selected items in our decision-tree analysis corroborated these findings. Moreover, all items from our decision-tree model are easily measurable and widely used as fall risk factors in clinical and research fields; therefore, our model is acceptable for use in a wide variety of situations. In this study, the selected predictors differed between the decision-tree model and the logistic regression model; TUG and lower limb pain were included only in the decision-tree model, whereas knee osteoarthritis and gait speed were included only in the logistic regression model. Logistic regression analysis is based on linear regression, whereas decision-tree analysis is based on a nonlinear model, and therefore different combinations of fall predictors might be selected by each model. Thus, the decision-tree analysis, with its nonlinear algorithm, may be useful in revealing stratified relationships between each fall predictor and subsequent fall risk.

As a feature point of our decision-tree model, an importance-based ordering of predictors is presented visually with respect to positions in the branch of the algorithm. Fall history is located at the top of the tree. Previous falls are known to be the most influential predictor [4], and the existing algorithm by the AGS/BGS Panel recommends assessing fall history first [9]. Therefore, we believe that the structure of our model is valid. Additionally, our decision tree calculated the optimal cut-off point of TUG as 9.7 s for fall prediction. Regarding the cut-off point of TUG for falls, the 13.5 s previously reported by Shumway-Cook et al. [28] has been widely used, and a previous systematic review showed that published cut-off points of TUG for independent-living older persons varied between 8.1 and 16.0 s [32]. Our cut-off, 9.7 s, is relatively fast, and this may be because it was calculated among our subjects without fall history or polypharmacy. Therefore, for older people who live independently and have not had any recent falls, the cut-off point of TUG for fall prediction might have to be set as a relatively faster time than previously believed.

The decision-tree model in this study outperformed the logistic regression model with respect to AUC, accuracy, sensitivity, PPV, and NPV. Although only specificity was lower in the decision-tree model than in the logistic regression model, the decision-tree model demonstrated relatively high sensitivity which would still make it suitable for use as a primary screening tool for fall risk. Additionally, our decision-tree model consists of common and easily measurable fall predictors and thus provides a minimal and personalized combination of predictors to calculate fall probability, ensuring that it can be useful as an efficient and effective tool in various healthcare settings.

A major strength of this study is that we analyzed large-scale, well-characterized cohort data using a longitudinal design. Additionally, we created a white-box decision-tree model using common fall risk factors without complicated calculations; thus, our fall-prediction model can be successfully applied to a variety of situations. However, this study has some limitations. First, our decision-tree model was only based on items assessed in medical or physical contexts; therefore, further examination combined with other aspects of fall risk should be conducted to improve the prediction performance. Second, although we used a cross-validation method, we did not use a hold-out dataset. Therefore, overfitting may still occur, and our results should be further validated in other external cohorts that have similar characteristics to the one used in the present study. Third, participants were healthy enough to undergo health checkups at the community center, and still 37.9% of participants dropped out of the follow-up survey. This selection bias may have led to an underrepresentation of baseline fall risk factors and future falls.

## 5. Conclusions

In this study, we developed a simplified decision-tree algorithm for fall prediction and confirmed its prediction validity using longitudinal cohort data. The decision-tree model outperformed the logistic regression model using the same predictors and could stratify the probabilities of fall incidence into various ranges. Our findings provide useful information for the early screening of fall risk and promote timely preventive strategies in community and clinical settings.

## Figures and Tables

**Table 1:** Baseline characteristics according to fall status in the follow-up survey.

Characteristics		Overall *n* = 2520	Non-Fallers *n* = 2105	Fallers *n* = 415	*p*-Value *
Age	(years)	71.1 ± 4.7	70.9 ± 4.6	71.8 ± 5.1	0.001
Female	(n, %)	1303 (51.7)	1072 (50.9)	231 (55.7)	0.078
Fall history	(n, %)	329 (13.1)	218 (10.4)	111 (26.7)	<0.001
Fear of falling	(n, %)	1051 (41.7)	838 (39.8)	213 (51.3)	<0.001
Prescribed medications	(drugs)	1.8 ± 1.9	1.8 ± 1.9	2.1 ± 2.2	0.002
Knee osteoarthritis	(n, %)	346 (13.7)	271 (12.9)	75 (18.1)	0.005
Lower limb pain	(n, %)	531 (21.1)	425 (20.2)	106 (25.5)	0.015
Gait speed	(m/s)	1.31 ± 0.20	1.32 ± 0.19	1.28 ± 0.21	<0.001
Timed up and go test	(s)	8.3 ± 1.5	8.3 ± 1.5	8.5 ± 1.7	0.007

Data are expressed as the mean ± standard deviation or numbers (%). * Based on Student’s *t*-test for continuous variables and χ^2^ tests for categorical variables.

**Table 2:** ORs of potential predictors for falls.

All Potential Predictors		Crude Model *	
		OR	95% CI
Age group			
<75 years		reference	
≥75 years		1.59	1.26−2.01
Sex			
Male		reference	
Female		1.21	0.98−1.50
Fall history			
No		reference	
Yes		3.16	2.44−4.09
Fear of falling			
No		reference	
Yes		1.59	1.29−1.97
Polypharmacy (≥ 5 drugs)			
No		reference	
Yes		1.88	1.37−2.57
Knee osteoarthritis			
No		reference	
Yes		1.49	1.13−1.98
Lower limb pain			
No		reference	
Yes		1.36	1.06−1.73
Gait speed	(m/s)	0.31	0.18−0.53
Timed up and go test	(s)	1.10	1.03−1.18

OR, odds ratio; CI, confidence interval. * Each item is set as an independent predictor in each separate model.

**Table 3:** Backward stepwise logistic regression model to predict falls.

Selected Predictors		Multivariable Model *	
		OR	95% CI
Age group			
<75 years		reference	
≥75 years		1.28	1.10−1.50
Fall history			
No		reference	
Yes		2.92	2.46−3.47
Fear of falling			
No		reference	
Yes		1.37	1.21−1.56
Polypharmacy (≥5 drugs)			
No		reference	
Yes		1.64	1.34−2.01
Knee osteoarthritis			
No		reference	
Yes		1.26	1.05−1.50
Gait speed	(m/s)	0.55	0.39−0.76

OR, odds ratio; CI, confidence interval. * All selected items are set as predictors simultaneously in single model.

**Table 4:** Model performance of logistic regression and decision tree for fall prediction.

Models	AUC	Accuracy	Sensitivity	Specificity	PPV	NPV
Logistic regression (Stepwise)	0.64 (0.63−0.66)	0.62 (0.60−0.63)	0.50 (0.48−0.52)	0.73 (0.71−0.75)	0.65 (0.63−0.68)	0.59 (0.58−0.61)
Decision tree (C5.0)	0.70 (0.68−0.72)	0.65 (0.64−0.67)	0.62 (0.60−0.64)	0.69 (0.67−0.71)	0.66 (0.64−0.69)	0.64 (0.62−0.66)

The values in brackets represent 95% confidence intervals. AUC, area under the curve; PPV, positive predictive value; NPV, negative predictive value.

## Data Availability

Due to participant confidentiality, only data presented in the paper are available.
